# Longitudinal analysis of individual cfDNA methylome patterns in metastatic prostate cancer

**DOI:** 10.1186/s13148-021-01155-w

**Published:** 2021-08-28

**Authors:** Romina Silva, Bruce Moran, Anne-Marie Baird, Colm J. O’Rourke, Stephen P. Finn, Ray McDermott, William Watson, William M. Gallagher, Donal J. Brennan, Antoinette S. Perry

**Affiliations:** 1 Cancer Biology and Therapeutics Laboratory, UCD Conway Institute of Biomolecular and Biomedical Research, University College Dublin, Dublin, Ireland; 2 School of Medicine, University College Dublin, Dublin, Ireland; 3 School of Biology and Environmental Science, Science West, O’Brien Science Centre, University College Dublin, Dublin, Ireland; 4 Department of Pathology, St. Vincent’s University Hospital, Dublin, Ireland; 5 Department of Clinical Medicine, Trinity College, Dublin, Ireland; 6 Biotech Research and Innovation Centre, Department of Health and Medical Sciences, University of Copenhagen, Copenhagen, Denmark; 7 Department of Histopathology, St James’s Hospital, Dublin, Ireland; 8 Cancer Trials Ireland, Dublin, Ireland; 9 Department of Medical Oncology, St. Vincent’s University Hospital, Dublin, Ireland; 10 School of Biomolecular and Biomedical Science, University College Dublin, Dublin, Ireland

**Keywords:** Metastatic prostate cancer, DNA methylation, Longitudinal study, cfDNA, Liquid biopsy

## Abstract

**Background:**

Disease progression and therapeutic resistance are hallmarks of advanced stage prostate cancer (PCa), which remains a major cause of cancer-related mortality around the world. Longitudinal studies, coupled with the use of liquid biopsies, offer a potentially new and minimally invasive platform to study the dynamics of tumour progression. Our aim was to investigate the dynamics of personal DNA methylomic profiles of metastatic PCa (mPCa) patients, during disease progression and therapy administration.

**Results:**

Forty-eight plasma samples from 9 mPCa patients were collected, longitudinally, over 13–21 months. After circulating cell-free DNA (cfDNA) isolation, DNA methylation was profiled using the Infinium MethylationEPIC BeadChip. The top 5% most variable probes across time, within each individual, were utilised to study dynamic methylation patterns during disease progression and therapeutic response. Statistical testing was carried out to identify differentially methylated genes (DMGs) in cfDNA, which were subsequently validated in two independent mPCa (cfDNA and FFPE tissue) cohorts. Individual cfDNA global methylation patterns were temporally stable throughout the disease course. However, a proportion of CpG sites presented a dynamic temporal pattern that was consistent with clinical events, including different therapies, and were prominently associated with genes linked to immune response pathways. Additionally, study of the tumour fraction of cfDNA identified > 2000 DMGs with dynamic methylation patterns.

**Conclusions:**

Longitudinal assessment of cfDNA methylation in mPCa patients unveiled dynamic patterns associated with disease progression and therapy administration, thus highlighting the potential of using liquid biopsies to study PCa evolution at a methylomic level.

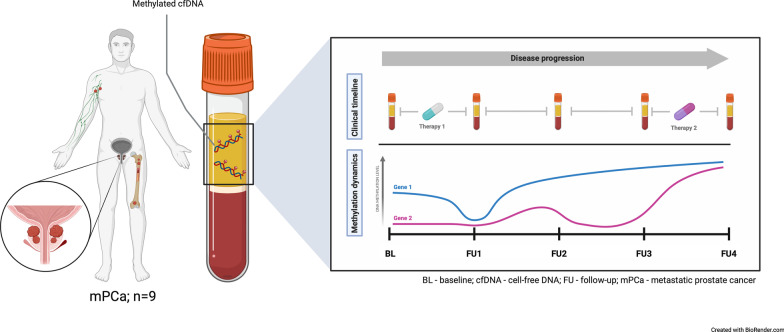

**Supplementary Information:**

The online version contains supplementary material available at 10.1186/s13148-021-01155-w.

## Introduction

Despite its 5-year survival rate of nearly 100% for localized disease, prostate cancer (PCa) is still considered a major cause of morbidity and mortality, accounting for approximately 7.6% of all cancer-related deaths worldwide [1]. Numerous studies have highlighted the importance of epigenetic aberrations, particularly DNA methylation, in PCa [2–4]. However, a knowledge-gap surrounds the dynamics of epigenetic alterations during disease progression and treatment resistance. Such investigations are typically hampered by the validity of performing molecular characterisation on archival tumour tissue acquired at diagnosis, often several years before disease progression. Moreover, acquiring serial biopsies or sampling multiple lesions to study tumour dynamics remains impractical and unethical.

Longitudinal studies have long been used to decipher the temporal association of disease traits in an effort to elucidate specific biological pathways and better understand variability observed among individuals. However, there is a dearth of longitudinal epigenetic studies in cancer, as most studies have focused on associations between epigenetic modifications and aging [5, 6]. Liquid biopsies provide an opportunity to overcome challenges associated with tissue biopsies and incorporate longitudinal assessment into the management of cancer patients. Circulating tumour DNA (ctDNA) can be noninvasively evaluated in blood plasma and several studies have shown that PCa-specific methylation alterations can be studied using this approach [7–9], with recent evidence showing that ctDNA profiles are consistent with metastatic tissue [10]. Additionally, since ctDNA is shed from different focal lesions, it overcomes spatial and clonal heterogeneity [11], thus making liquid biopsies an ideal surrogate to study the dynamics of tumour progression and real-time disease monitoring.

The aim of this study was to investigate the dynamics of individual methylomic profiles of metastatic PCa (mPCa) patients. Through longitudinal analysis of circulating cell-free DNA (cfDNA), we demonstrate the feasibility of this approach to study tumour dynamics and monitor disease-associated characteristics.

## Results

### Study cohort characteristics

Longitudinal assessment of methylation dynamics in 9 mPCa patients (comprising 48 cfDNA samples) was performed over at least 4 time points for an average of 19.11 months (Table 1). The shortest follow-up time (13 months) was attributed to death from disease. During follow-up, subjects received up to four different therapies: 88.9% had luteinizing hormone-releasing hormone (LHRH) agonists/antagonists, 100% had chemotherapy (taxanes), 88.9% had androgen receptor (AR) inhibitors and 33.3% had radioisotopes.

**Table 1:** Clinical characteristics of the study cohort (*n* = 9)

Mean age^a^, years (range)	64.2 (51–74)
*Gleason score at diagnosis*^*b*^*, n (%)*	
7 (3 + 4)	5 (55.6)
9	4 (44.4)
*Metastatic sites, n (%)*	
Bone	9 (100)
Lymph node	2 (22.2)
*Evidence of castration resistance at recruitment (cohort)*^*c*^*, n (%)*	
No (mPCa; cohort 1)	5 (55.6)
Yes (mCRPC; cohort 2)	4 (44.4)
*Number of timepoints*^*d*^*, n (%)*	
4	2 (22.2)
5	2 (22.2)
6	5 (55.6)
Mean cfDNA concentration^e^, ng/ul (range)	5.08 (0.61–38)
Mean PSA levels^e^, ng/ml (range)	35.3 (0.1–352.1)
*Therapies administered*^*f*^*, n (%)*	
LHRH agonists/antagonists	8 (88.9)
Taxanes	9 (100)
AR-inhibitors	8 (88.9)
Radioisotopes	3 (33.3)
Mean follow-up time^g^, months (range)	19.1 (13–21)

*AR* androgen receptor, *cfDNA* cell-free DNA, *LHRH* Luteinizing hormone-releasing hormone, *mPCa* metastatic prostate cancer, *mCRPC* metastatic castration resistant prostate cancer, *PSA* prostate-specific antigen

Other notes: ^a^Age at time of recruitment

^b^Prior to recruitment

^c^Cohort classification: Cohort 1—patient was on/starting androgen deprivation therapy at time of recruitment, and had no evidence of castration resistance; Cohort 2—patient had castration resistant disease at time of recruitment

^d^Timepoint refers to number of cfDNA isolations available for study

^e^At any given timepoint

^f^During follow-up in the iPROSPECT study

^g^Follow-up time reflects last timepoint obtained for this analysis

**Fig. 1:**
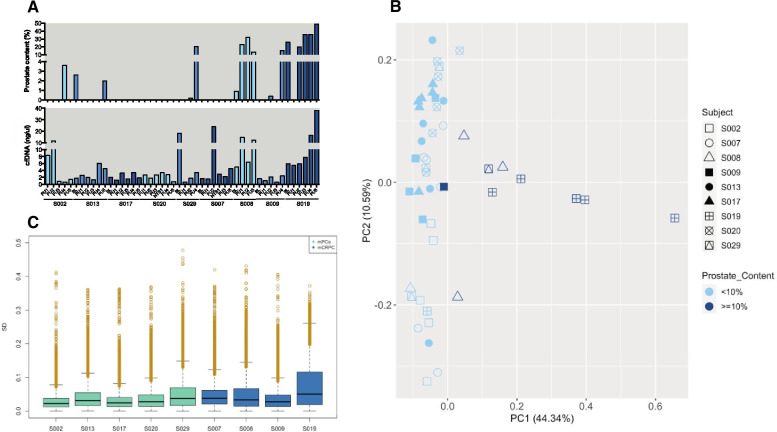
Longitudinal assessment of global methylation uncovers low-intra subject variability. **A** cfDNA concentration (ng/µL) and proportion of prostate content (%) for forty-eight cfDNA samples. cfDNA concentration ranged from 0.61 to 38 ng/µL and prostate content from 0 to 49.1%. **B** Principal component analysis of the 801,849 EPIC array probes available after QC and pre-processing of raw data depicts the methylation differences between the 9 subjects at different timepoints. **C** Temporal probe variability for each subject. Gold circles represent the top 5% most variable probes. *BL* Baseline, *cfDNA* cell-free DNA, *FU* follow-up, *mCRPC* metastatic castration resistant prostate cancer, *mPCa* metastatic prostate cancer, *NTS* new treatment strategy, *SD* standard deviation

To assess the prostate content in the cfDNA samples, we utilised the *human cell-type DNA methylation atlas* [12]. Sixteen of 48 samples (33.3%), across 6 patients, had detectable prostate-derived DNA; 10 of whom had prostate content (PC) ≥ 10% (Fig. 1A). Samples with the largest prostate contribution came from two subjects, one (S019) had the highest tumour burden (as measured by prostate-specific antigen, PSA), and the second (S008) died from disease during follow-up. For the remaining samples, sources of cfDNA were largely attributed to cells of hematopoietic origin, as previously observed [12].

### Global cfDNA methylation profiles are temporally stable

In order to understand the dynamics of cfDNA methylation during PCa progression and sequential therapies, we analysed the global methylation patterns of all samples. Principal component analysis was used to understand methylation differences between individuals, with this analysis showing little variance between samples and/or individuals (Fig. 1B). Interestingly, the biggest difference stemmed from prostate cellular contribution, as samples from S019 clearly separated from all other samples. We next investigated the degree to which global methylation changed over time within individuals. Evaluation of temporal divergence showed that most probes remained relatively stable (standard deviation—SD < 0.1), indicating a low intra-subject variation in cfDNA methylation (Fig. 1C). Again, S019 was distinct, displaying the greatest degree of temporal variation.

**Fig. 2:**
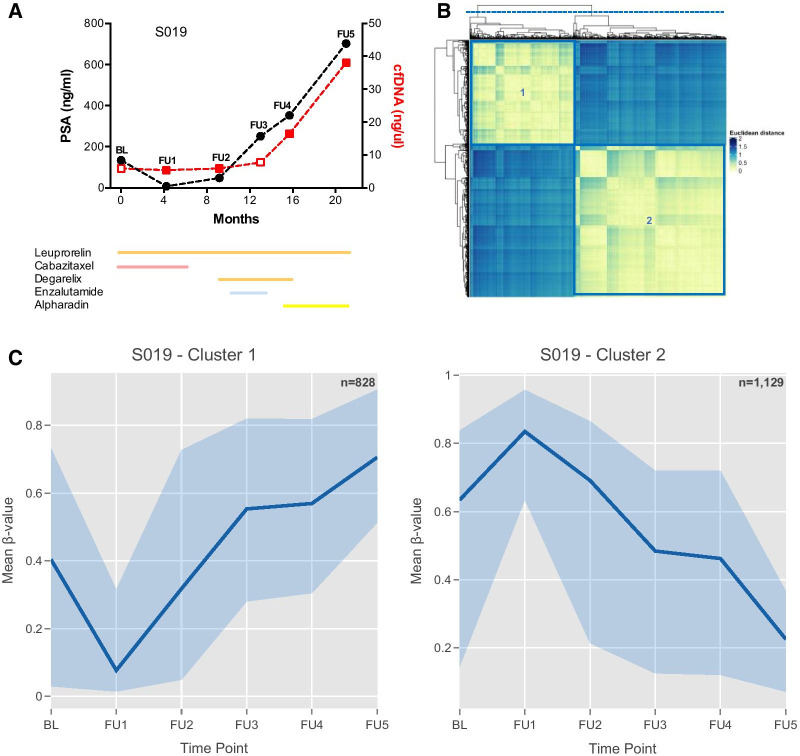
Longitudinal analysis of metastatic PCa patient S019 highlights dynamic methylation patterns across time and their association with clinical findings. **A** Disease course (since recruitment to iPROSPECT study—baseline). PSA levels (ng/mL) and cfDNA concentration (ng/µL) are represented in the left (black) and right (red) *y* axis, respectively. Details of therapies administered are indicated by coloured lines below the graph: LHRH agonists/antagonist (orange), taxane (pink), AR inhibitors (blue) and radioisotopes (yellow). **B** Heatmap showing the similarity of patterns between probes, located in 5′ regulatory regions (*n* = 9549), across time. Probes with similar methylation patterns were aggregated into clusters, which are identified in the heatmap by purple boxes and numbers. Unsupervised clustering was performed using euclidean distances between probes and coloured legend reflects magnitude of those distances (blue to yellow–small to large distance). **C** Methylation dynamics observed for genes identified through cluster analysis, with total number of genes used indicated in the top right corner for each cluster. Only genes represented by ≥ 2 dynamic CpGs are shown. Blue filled area represents methylation values observed at each time point, with edges indicating the maximum and minimum values observed. Darker blue line represents the observed mean methylation value of all genes. Black lines, on the upper part of the plots, indicate the duration of administration of a specific PCa therapies, whose effect was explored in our study (taxanes—T; AR inhibitors—AR). *BL* baseline, *cfDNA* cell-free DNA, *FU* follow-up, *PSA* prostate specific antigen

The dynamic patterns of the 5′-regulatory probes within the top 5% most variable probes (MVPs) (Additional file 1: Fig. S1) were analysed for congruence over time, and assessed in conjunction with the disease course for each individual (Fig. 2A and Additional file 1: Figs. S2–S9). Probes with similar patterns were aggregated into clusters. Individuals displayed as few as two probe clusters (S019) and as many as seven (S007) (Fig. 2B and Additional file 1: Figs. S2–S9). Greater numbers of probe clusters suggest that the most variably methylated genes in these patients behave differently throughout the disease course and therapeutic response. Indeed, some methylated gene clusters correlated with clinical events. For example, in subjects with high PC samples (S008 and S019), methylation dynamics mimicked PSA patterns, with methylation shifts coinciding with PSA changes observed in these patients (Fig. 2 and Additional file 1: Fig. S8). Findings also indicate that therapy administration may affect cfDNA methylation dynamics, with most subjects demonstrating a notable shift in methylation patterns following taxane administration (Fig. 2C and Additional file 1: Figs. S2–S9). This is evident, for example, by the methylation difference observed in S019 between administration of cabazitaxel at baseline (BL) and follow-up 1 (FU1) (Fig. 2C). AR inhibition may also confer temporal epigenomic shifts, as shown by methylation changes in 4/6 probe clusters in S007, after commencing enzalutamide at month 11 (Additional file 1: Fig. S7).

Overall, although the majority of the methylome remained stable over time, a proportion of 5′ regulatory CpGs had a dynamic longitudinal pattern, consistent with clinical events.

### Dynamic methylation patterns during therapy administration are associated with an immune response element

**Fig. 3:**
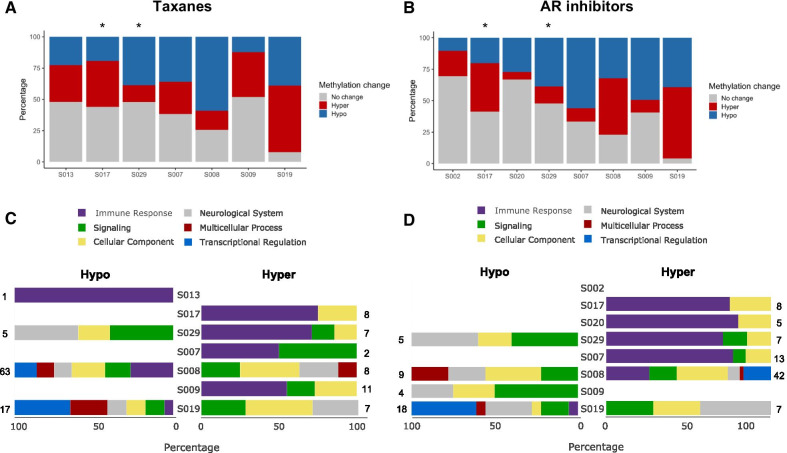
Dynamic methylation patterns observed during PCa therapy administration and their biological relevance. Stacked bar graphs for **A** taxanes and **B** AR inhibitors, indicate the proportion of the top 5% most variable probes (located in 5′ regulatory regions) with an absolute methylation change ≥ 0.1 between the time-points before and after therapy administration. Probes are classified as hyper- or hypomethylated according to whether the change resulted in a gain or loss of methylation, respectively. Subjects for whom these two therapies were administered simultaneously (*) are indicated. Gene ontology (GO) analysis of probes with methylation changes that were consistent with administration of **C** taxanes and **D** AR inhibitors. Total number of significant GO terms, for each subject and each dataset (hyper and hypo) is indicated in the right and left edges of the graph. Significance threshold was set at FDR *p* value < 0.05

To further explore the relationship between cfDNA methylation changes and therapy administration, we examined each subject’s MVPs and measured methylation changes (absolute *β* ≥ 0.1 difference) between time-points after therapy began. For most subjects, over 50% of the MVPs displayed a methylation change after taxane (Fig. 3A) or AR inhibitor (Fig. 3B). Notably, both drugs were administered simultaneously for S017 and S029, hindering any conclusions about methylation changes that might be associated with a specific therapy.

A detailed look at the cluster analysis also uncovered a methylation shift after taxane termination, such as the ones observed after FU1 in S019, bringing methylation patterns to a pre-therapy level (Fig. 2C; other examples are shown in Additional file 1: Figs. S2–S9). Indeed, reviewing all hyper- and hypo-MVPs showed that most were only transiently affected by taxanes, with their methylation shift largely dissipating upon therapy cessation (Additional file 1: Fig. S10).

Additionally, we investigated if the probes that changed after therapy administration were associated with any biological processes and pathways. Studying the most significantly represented processes associated with both taxane and AR therapy administration (FDR *p* < 0.05), we observed that a high proportion of gene ontology (GO) terms were associated with immune response (Fig. 3C, D). Other processes were also enriched, such as signaling, cellular components and neurological system. This might be expected, since most cfDNA samples had substantial contribution from cells of hematopoietic origin. Notably, S008 and S019 were distinct; their cfDNA was enriched for transcriptional regulation and multicellular processes, perhaps reflecting their higher PC and thus genes involved in PCa (Fig. 3C, D).

### ctDNA temporal dynamics in mPCa patients

**Fig. 4:**
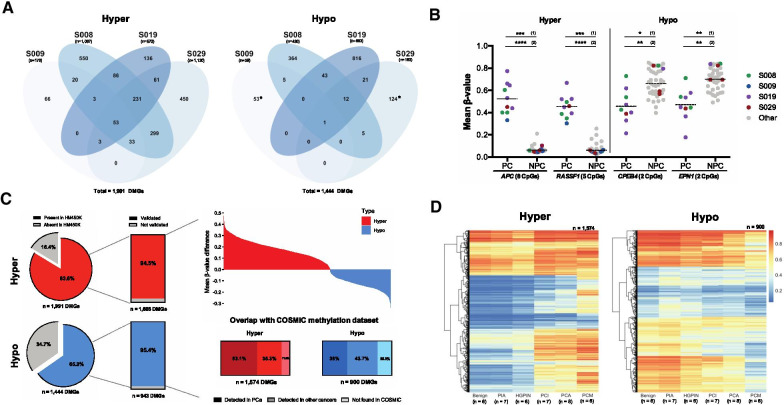
Identification of differentially methylated genes in cfDNA samples with prostate content and validation of results across independent cohorts. **A** Numbers of significantly hyper and hypomethylated differential methylated genes (DMGs) identified by comparing samples with prostate cell content (PC) versus samples without (NPC), across the 4 subjects with samples having substantial prostate content (≥ 10%). **B** Schematic representation of 4 DMGs. Circles represent individual mean *β*-values. The 4 subjects with PC samples are distinguished from the 5 remaining subjects (grey). Statistical analysis identified differences between (1) PC and NPC samples within subjects with prostate content and (2) PC samples from subjects with prostate content and all NPC samples. **C** Validation of DMGs in an independent cfDNA cohort (*n* = 181). Waterfall plot indicates the degree of methylation difference between NPC (considered baseline in this plot) and PC samples for validated DMGs. Additionally, overlap of DMGs and the COSMIC methylation dataset indicated genes whose methylation had previously been detected in PCa and/or other cancers. **D** Heatmap showing methylation status of validated DMGs in an independent PCa tissue cohort, comprising all histological stages of PCa development. Differences between PC and NPC samples in **B** and **C** were carried out using an independent *t* test/Mann–Whitney *U* test, and multiple testing correction (Benjamini–Hochberg method) was used when appropriate. *p* values are as follow: *p ≤ 0.05; **p ≤ 0.01; ***p ≤ 0.001; ****p ≤ 0.0001. *PC* prostate content, *NPC* no prostate content, *PIA* proliferative inflammatory atrophy, *HGPIN* high-grade prostatic intraepithelial neoplasia, *PCI* prostate cancer (indolent), *PCA* prostate cancer (aggressive), *PCM* prostate cancer (metastatic)

Irrespective of therapy administration, we also examined the tumour fraction of cfDNA methylation patterns. To do this, we used the top 5% promoter-associated MVPs to identify genes with dynamic methylation patterns among subjects with PC samples (S008, S009, S019, S029). After assessment of their methylation values across samples with no prostate content (NPC) from the whole cohort (to verify their ctDNA-specificity), a list of 3435 differentially methylated genes (DMGs) was compiled, 1991 hyper- and 1444 hypomethylated (Fig. 4A). Notably, several DMGs are already known to be involved in PCa carcinogenesis. For example, in our cohort, both *APC* and *RASSF1* demonstrated higher methylation, and *CPEB4* and *EPN1* showed lower methylation, when comparing PC versus NPC samples (Fig. 4B). The majority (72%) of DMGs (1574 hyper- and 900 hypomethylated) validated in an independent mPCa cfDNA cohort, where DMGs were also compared between PC and NPC samples [13] (Fig. 4C). Additionally, overlap with the publicly-available COSMIC methylation dataset, showed that methylation changes in 53% and 36% of these hyper and hypomethylated genes, respectively, has been previously implicated in PCa (Fig. 4C).

Further validation was carried out in a formalin-fixed, paraffin-embedded (FFPE) tissue cohort of six distinct stages of PCa, to account for potential confounding cfDNA methylation originating from non-prostate sources. Certain hyper- and hypomethylated DMGs show similar methylation patterns across PCa stages, which might suggest that these DMGs are prostate-specific and differences can only be seen when comparing prostate with other tissues/cfDNA sources. However, the methylation values for most mPCa DMGs were similar to observations in the metastatic stage of the FFPE cohort, validating our results and suggesting an association of these epigenetic alterations and the more advanced stages of PCa (Fig. 4D). Additionally, methylation differences were observed across the different stages of PCa, emphasizing the potential role of DNA methylation in disease progression (Fig. 4D and Additional file 1: Fig. S11).

The majority of both hyper and hypomethylated DMGs (60.37% and 93.98%, respectively) were notably unique to one subject (Fig. 4A). This again demonstrates the renowned molecular heterogeneity of PCa, and the need for personalised medicine approaches such as this.

## Discussion

In this study, we describe the first personal methylomic longitudinal analysis of PCa using cfDNA from 9 mPCa patients. Our results reveal that distinct methylation dynamics, observed in a proportion of assessed CpG sites, were consistent with different clinical events, such as PSA progression and therapy administration. Additionally, specifically studying ctDNA methylation dynamics allowed us to identify genes previously observed as epigenetically modified in mPCa [2, 7, 14].

Longitudinal studies are defined by following individuals over time to identify disease-specific temporal patterns that relate to stage or burden of disease, or response to a given therapy. Understanding these specific patterns may result in novel diagnostic, prognostic and monitoring tools that could improve clinical management, and also help reduce the burden on the healthcare system. This is particularly relevant for advanced stage PCa, as the lack of detailed knowledge surrounding disease progression has affected clinical management. The standard use of PSA as a biomarker has been of great benefit for monitoring disease outcomes in PCa (i.e. disease progression and response to therapy) [15]. However, it offers no insight into the molecular mechanisms driving tumour progression or resistance to therapy. Thus, there is an unmet need to investigate PCa-specific temporal patterns that might uncover the foundations of disease progression and contribute towards the establishment of a biomarker-guided and risk stratification-based clinical management of PCa.

We employed an epigenome-wide array to understand longitudinal methylation patterns and how these might be influenced by disease progression and treatment change in men with metastatic PCa. As previously reported [8, 12], the use of array platforms with liquid biopsy samples averages DNA methylation from distinctive, and potentially multiple, cell types present. We therefore employed a methylation atlas to identify the source(s) of the main cellular components in each subject’s cfDNA [12]. One-third of the cfDNA samples in our cohort had detectable prostate content; a frequency on par with previous mPCa cfDNA studies [16–18]. While statistical methods have been developed that adjust methylation data for cell-type heterogeneity [19], most focus on whole blood cell populations or more generic cell types (i.e. epithelial cells), overlooking the necessity to analyse tumour-specific patterns. Additionally, there is a dearth of statistical methods for *n*-of-1 or personalised approaches, such as this. Applying these approaches in this study could affect their robustness and lead to erroneous adjustments. Thus, the longitudinal observations made in this study are largely related to the total cfDNA content.

Temporal analysis of liquid biopsy DNA methylation patterns in mPCa individuals throughout disease progression, drug treatment(s) and relapse showed that most CpG sites did not substantially change. This finding supports previous research, which demonstrated that the human methylome is relatively stable over time [5, 20, 21]. However, the proportion of the methylome that demonstrated temporal variation was notably evident on administration of two PCa therapies: taxanes and AR inhibitors. To the best of our knowledge this is the first study to interrogate how therapies might affect the PCa methylome over time. Changes in CpG methylation following therapy administration were most pronounced in genes involved with immune response, including ones that encode essential components of the JAK-STAT pathway, an important regulator of the inflammatory response [22]. This discovery is bolstered by previous reports, which showed that chemotherapy-induced methylation changes could influence immune cells and pathways [23, 24]. We also observed that this potential therapy-induced methylation shift was transient, and was only typically present during the time of drug administration. We suggest that this ephemeral pattern may be indicative of a systemic-epigenomic response to chemotherapy, rather than an epigenetic reprogramming, as previously observed during PCa progression [25], which would be unlikely to disappear after treatment cessation. Conversely to systemic treatments, AR inhibitors are a targeted PCa therapy that function by antagonising AR signalling [26]. Although AR receptors are mostly expressed in reproductive tissues, they have also been implicated in the regulation of multiple cellular processes in a variety of other organs and systems [27, 28]. In fact, recent reports suggest that AR inhibitors might have a role in modulating the immune response in PCa [29, 30]. However, the molecular mechanisms by which AR inhibitors exert this immune-effect are still unknown. Our findings suggest that an epigenetic component could be involved.

Longitudinal assessment of methylation patterns allowed us to explore the dynamics of epigenetic events during metastatic PCa. Using our cohort as a discovery set and validating in two independent cohorts, we identified 2474 DMGs, with some (i.e. *AR*, *GSTP1*, *RASSF1*, *APC*) previously linked to PCa [2]. Two noteworthy hypomethylated genes are *EPN1* and *CPEB4*. EPN1 is known to actively promote tumorigenesis by enhancing cell surface receptor endocytosis and upregulating tumour growth-related pathways [31]. CPEB4, an mRNA binding protein, actively reprogrammes gene expression by acting as a translational repressor/activator and promoting an invasive phenotype [32]. While elevated expression of both genes has been reported in several invasive cancers, including prostate [31, 33], the mechanism of their upregulation was previously unknown. Here, we show that their promoter regions are hypomethylated in cfDNA (with detectable prostate content) from mPCa patients, suggesting that a loss of promoter methylation might play a role in the upregulation of *CPEB4* and *EPN1* in advanced PCa.

Individualised longitudinal studies, such as this, are important for precision medicine and to enable a better understanding of how to tailor clinical decisions [34, 35]. Indeed, by analysing serial plasma samples over the course of 36 months, Chen and colleagues were able to demonstrate that personal DNA methylomes showed distinct dynamic patterns that were associated with different physiological conditions, and that those patterns were present before the onset of the disease [34]. Our study does have some limitations. As an observational study with a small cohort size, we cannot causally attribute methylation changes to specific therapies and any attempt to do so would be an over interpretation of the data. Further studies are needed to validate our findings and provide robust translational evidence for the application of this methodology to the clinic. We prioritised promoter methylation within the top 5% MVPs in each subject, in order to study the most temporally variable probes and focus on biologically relevant regions, as promoter methylation is highly correlated with gene expression. However, this represents only ~ 1% of CpG sites represented on the EPIC array and > 0.04% of all human CpGs. A deeper dive into how the methylome (beyond the conventional promoter CpG island) evolves during disease progression and therapeutic resistance is warranted.

## Conclusions

Overall, we show the feasibility of studying longitudinal cfDNA methylation patterns in individuals and demonstrate how these profiles might be influenced by clinical events, such as therapeutic administration. Further analysis into the identification of PCa-specific temporal patterns might improve our understanding on how to better use cfDNA-methylation changes for improving monitoring and clinical management of this malignancy.

## Methods

### Clinical cohorts

Patients were recruited between 2015 and 2016, to a Cancer Trials Ireland sponsored translational, multicentre, longitudinal study of men with mPCa titled CTRIAL-IE (ICORG) 14-04 Irish Programme for Stratified Prostate Cancer Therapy (iPROSPECT). All patients gave informed consent for serial blood sampling.

Additionally, two independent validation cohorts were used: (1) an FFPE tissue cohort of different histological stages of PCa (*n* = 44, Additional file 1: Table S1 and GSE157272), (2) and a previously described cfDNA dataset (*n* = 181 mPCa patients) [13].

### Sample collection and DNA methylation profiling

**Fig. 5:**
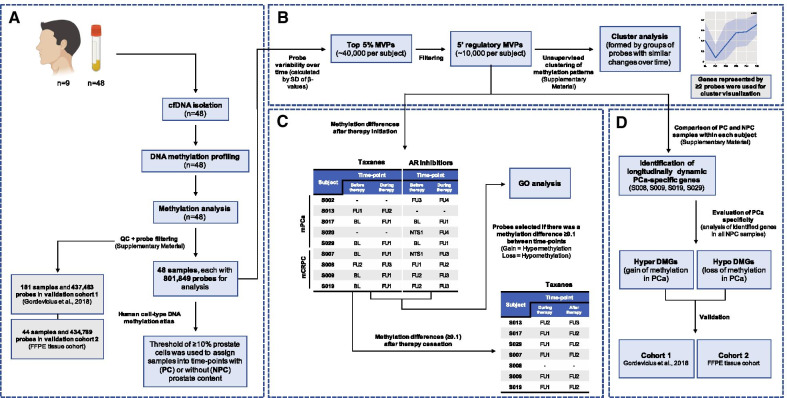
Overview of patient samples and study methods. **A** Fifty-two plasma samples were used for cfDNA isolation and DNA methylation profiling, 48 of which were further analysed. Raw data were pre-processed and were characterised for cellular origin. **B** The top 5% most variable probes (MVPs) within each subject over time were filtered for 5′ regulatory regions and used to analyse dynamic methylation patterns, generating congruent clusters. **C** The effects of drug therapy on cfDNA methylation were analysed by examining changes in methylation of ≥ 0.1 (between drug time-points). **D** Methylation differences between PC and NPC samples were analysed for the 4 subjects with any number of PC samples. Identified differentially methylated genes (DMGs) were validated using two independent PCa cohorts. *BL* baseline, *cfDNA* cell-free DNA, *DMGs* differentially methylated genes, *FU* follow-up, *mCRPC* metastatic castration resistant prostate cancer, *mPCa* metastatic prostate cancer, *MVPs* most variable probes, *NPC* non-prostate content, *PC* prostate content, *SD* standard variation

Peripheral blood samples were collected at baseline (BL), and subsequent follow-ups (FU), every 4 ± 1 months. Plasma was isolated, by centrifugation at 2000×*g* for 15 min, within 2 h of collection and stored at – 80 °C. Forty-eight plasma samples (≤ 3 mL) from 9 participants across multiple time points were used for cfDNA isolation, as previously described [8]. cfDNA was bisulfite modified using the Zymo EZ DNA methylation kit and quality control was by real-time PCR quantification, as previously described [36]. Bisulfite modified DNA was subjected to the Illumina Restoration kit and analysed using the Illumina Infinium Human MethylationEPIC BeadChip platform (Fig. 5A). Raw data (GSE157273), were pre-processed using *minfi* [37]. After QC, normalization and probe filtering (Additional file 1), a total of 801,849 probes were retained for analysis. A similar data processing pipeline was applied to the two validation cohorts, both run on the Illumina HM450K platform.

The *human cell-type DNA methylation atlas* [12] was used to determine tissue origin of cfDNA samples and classify samples into those with prostate content (PC) or without (NPC) (Fig. 5A).

### Longitudinal methylation analysis

The top 5% MVPs within each subject over time (calculated by SD of *β*-values), were used to study dynamic methylation patterns. Further analysis concentrated on 5′ regulatory probes, as characterised in Illumina’s EPIC array manifesto (TSS1500, TSS200 and 5′UTR), which were used to identify clusters of probes that had similar methylation patterns over time [38] (Fig. 5B and Additional file 1). Additionally, the effects of different therapies, taxanes and AR inhibitors, on the methylation of cfDNA were examined using the top 5% MVPs between commencement and cessation of therapy (Fig. 5C and Additional file 1). GO was performed as previously described [39, 40]. Finally, ctDNA dynamics were investigated by analysing genes identified in subjects with PC cfDNA (S008, S009, S019 and S029). Genes were considered differentially methylated (DMGs) after comparison with NPC samples from the whole dataset (*β* ≥ 0.1 difference, adjusted *p* value < 0.05) (Additional file 1). Validation of DMGs was carried out using two independent PCa cohorts (Fig. 5D).

### Statistical analysis

Unpaired t-test/Mann–Whitney *U* test were used to identify DMGs to study ctDNA dynamics; Kruskal–Wallis and Dunn’s multiple comparison tests were used to evaluate methylation across different histological stages of PCa carcinogenesis. Analyses were performed using Prism 6 (GraphPad) and R (v3.6.3) and deemed significant if FDR-adjusted *p* value < 0.05. The Benjamini–Hochberg method was used for multiple testing correction when appropriate.

## Supplementary Information

**Additional file 1.** File containing supplemental figures and tables necessary for the full comprehension of the manuscript.

## Data Availability

The datasets generated and/or analysed during the current study are available in the GEO repository (GSE157272, GSE157273).

## Abbreviations

AR: Androgen receptor
BL: Baseline
cfDNA: Cell-free DNA
ctDNA: Circulating tumour DNA
DMGs: Differentially methylated genes
FU: Follow-up
GO: Gene ontology
LHRH: Luteinizing hormone-releasing hormone
iPROSPECT: Irish Programme for Stratified Prostate Cancer Therapy
mPCa: Metastatic prostate cancer
MVPs: Most variable probes
NPC: Non-prostate content
PC: Prostate content
PCa: Prostate cancer
PSA: Prostate specific antigen
