# Supplementary material for: Longitudinal analysis of individual cfDNA methylome patterns in metastatic prostate cancer
Source: Clin Epigenetics. 2021 Aug 28;13:168. doi: 10.1186/s13148-021-01155-w (PMC8403420; doi:10.1186/s13148-021-01155-w)
Supplement: Supplementary file 1 — Additional file 1. File containing supplemental figures and tables necessary for the full comprehension of the manuscript. [file 13148_2021_1155_MOESM1_ESM.docx]

**SUPPLEMENTAL DATA**

**SUPPLEMENTAL METHODS**

**Data processing**

Raw data were imported into R for preprocessing using *minfi*(1). QC was performed at both the i) sample and ii) probe level. All 48 samples had a mean detection *P*-value <0.05 and no sample mix-ups were detected via analysis of 65 germline SNPs included in the array (**Supplemental Figure 12)**. Prior to probe QC, data were normalized using functional normalization with Noob background correction(2). 9,309 probes with a non-significant detection threshold (*P*>0.05) for 25% or more of the samples were excluded. The following probes were also excluded: probes with SNPs at the single base extension or with a SNP at the CpG site (minor allele frequency≥5%; n=11,272), non-CpG probes (n=2,909), and cross-reactive probes previously identified in the EPIC array (n=41,497)(3). As all study subjects were male, probes annotated to sex chromosomes were retained. This resulted in 801,849 probes for analyses.

A similar data processing pipeline was applied to the validation cohorts, both run on Illumina’s HM450K array, with a total availability of 434,789 probes for the FFPE tissue and 437,483 probes for the mPCa cfDNA cohorts (raw data obtained from GEO – GSE108462).

**Human Methylation Reference Atlas**

The human cell-type DNA methylation atlas (4) was used to determine the tissue origin of cfDNA molecules in each of the 48 samples measured on the array. Data preprocessing and specific cell-type methylation were performed following instructions by the authors (https://github.com/nloyfer/meth_atlas). Only time-points with ≥10% of prostate cells were designated as samples with prostate content (PC), while the other were designated as no prostate content (NPC). The same pipeline was used to characterize the mPCa cfDNA samples from the validation cohort.

**Longitudinal Methylation Patterns – Dynamic methylation patterns over time**

To study methylation patterns over time, the standard deviation (SD) in β-values was calculated for each probe across all time-points, for each individual subject. Within each subject, the top 5% most variable probes (MVPs) were selected for further analysis. To understand the association between changes in methylation and biological functions, probes were filtered for those found in 5’-regulatory regions. To identify congruent changes among the probes, probe patterns across time-points were compared using *TSdist* package(5). For this analysis, each probe (and its measurements across time) represents one single time series. Each series was compared to all others and a euclidean distance matrix was created, based on the similarity of probe patterns across time. Unsupervised clustering of this matrix was used to create clusters of probes that had similar patterns, and a determined numbered label was assigned to each cluster by the algorithm. From these clusters, genes represented by ≥2 probes were selected and a mean methylation value was calculated for each gene using β-values of selected probes located in that same gene. Mean values were used to plot each cluster and visualize the methylation patterns across time.

**Longitudinal Methylation Patterns – Therapy administration**

Occurrence of methylation changes with regards to therapy administration was evaluated for both taxanes and androgen receptor (AR) inhibitors for 7 and 8 subjects, respectively. In both instances, the top 5% MVPs located in promoters were used and a methylation value difference was calculated between the time-point before therapy administration started and the one immediately after (during therapy). For subjects who were administrated several drugs of the same group (S002 and S020 received both bicalutamide and enzalutamide), methylation difference was only calculated for the drug that had availability of the time-points above indicated (bicalutamide for S002; enzalutamide for S020). Probes with an absolute methylation difference of ≥0.1 were selected and identified as being hyper (methylation gain) or hypomethylated (methylation loss). Using identified hyper- and hypomethylated probes, gene ontology (GO) was performed with the *gometh* function (*missMethyl* package), in order to control for the significant bias associated with different genes having different number of probes mapping to them in arrays (6, 7). S002 and S020 were not included in the taxane analysis group due to lack of a time-point before the start of therapy administration.

Additionally, we inquired if there were any changes in the identified probes after cessation of taxane administration. This was done by calculating the methylation difference between the last time-point of therapy administration and the one after. The same threshold of ≥0.1 change was applied and changes were only validated if there was a methylation change in the opposite direction of the previously observed one (e.g. hypomethylated probe after start of therapy administration had to display hypermethylation after cessation). Of the subjects initially evaluated for methylation changes upon start of taxanes, only S008 was excluded due to lack of a time-point after therapy was finished.

**Methylation Analysis – ctDNA methylation dynamics**

Investigation of the ctDNA methylation dynamics was carried out by identifying genes with distinct methylation patterns among the 4 subjects with PC samples (S008, S009, S019, S029). Genes were identified within each subject first, by using the top 5% 5’ regulatory MVPs. For each probe, a mean methylation β-value observed in PC and NPC samples was calculated. Genes were then selected if (i) probes had ≥0.1 absolute methylation difference between PC and NPC samples, (ii) ≥2 probes mapped to the same gene. For any common genes across two or more of these subjects, the gene was only selected if probes were also common. These were separated in hyper and hypomethylated datasets, according to whether observed methylation difference was positive or negative, respectively. Finally, differentially methylated genes (DMGs) were chosen if the methylation differences observed between the 4 subjects’ PC samples (n=9) and all subjects’ NPC samples (n=39) was significant (independent t-test/ Mann-Whitney U; significance threshold: FDR *P*-value < 0.05). Genes uniquely identified on subjects S009 and S029 were not subjected to statistical analysis, as they only had one available PC time-point, but were still added to the DMG list. A Venn diagram, compiling all results, was created.

Validation of DMGs was carried out using an independent mPCa cfDNA cohort(8), where samples were characterised as PC (n=34) or NPC (n=147), following the same criteria used for our cohort. This cohort was obtained using HM450K array, so only DMGs with any number of matching probes in that array were evaluated. Statistical testing employed during validation was the same as in discovery cohort. Validated DMGs were visually represented by a waterfall plot, indicating the methylation difference between NPC and PC samples, and overlapped with the COSMIC methylation dataset (v.91)(9) to identify which DMGs had been previously detected in PCa or other cancers. Finally, using validated DMGs and an independent FFPE tissue cohort, we evaluated the methylation of these genes across several histological stages of PCa carcinogenesis. Methylation across the different stages was visualized with the help of a heatmap plot and statistical testing to evaluate differences was carried out for 3 of the DMGs (**Supplemental Figure 11**; Kruskal-Wallis, followed by Dunn’s multiple comparison test, with significance threshold of *P*-value < 0.05). Multiple testing correction (Benjamini-Hochberg method) was used when appropriate.

**REFERENCES:**

1. Aryee MJ, Jaffe AE, Corrada-Bravo H, Ladd-Acosta C, Feinberg AP, Hansen KD, et al. Minfi: a flexible and comprehensive Bioconductor package for the analysis of Infinium DNA methylation microarrays. Bioinformatics. 2014;30(10):1363-9.

2. Fortin JP, Labbe A, Lemire M, Zanke BW, Hudson TJ, Fertig EJ, et al. Functional normalization of 450k methylation array data improves replication in large cancer studies. Genome Biol. 2014;15(12):503.

3. Pidsley R, Zotenko E, Peters TJ, Lawrence MG, Risbridger GP, Molloy P, et al. Critical evaluation of the Illumina MethylationEPIC BeadChip microarray for whole-genome DNA methylation profiling. Genome Biol. 2016;17(1):208.

4. Moss J, Magenheim J, Neiman D, Zemmour H, Loyfer N, Korach A, et al. Comprehensive human cell-type methylation atlas reveals origins of circulating cell-free DNA in health and disease. Nat Commun. 2018;9(1):5068.

5. Mori U, Mendiburu A, Lozano J. TSdist: Distance Measures for Time Series Data. 2019; R package version 3.6.

6. Geeleher P, Hartnett L, Egan LJ, Golden A, Raja Ali RA, Seoighe C. Gene-set analysis is severely biased when applied to genome-wide methylation data. Bioinformatics. 2013;29(15):1851-7.

7. Phipson B, Maksimovic J, Oshlack A. missMethyl: an R package for analyzing data from Illumina's HumanMethylation450 platform. Bioinformatics. 2016;32(2):286-8.

8. Gordevicius J, Krisciunas A, Groot DE, Yip SM, Susic M, Kwan A, et al. Cell-Free DNA Modification Dynamics in Abiraterone Acetate-Treated Prostate Cancer Patients. Clin Cancer Res. 2018;24(14):3317-24.

9. Tate JG, Bamford S, Jubb HC, Sondka Z, Beare DM, Bindal N, et al. COSMIC: the Catalogue Of Somatic Mutations In Cancer. Nucleic acids research. 2018;47(D1):D941-D7.

**SUPPLEMENTAL TABLES AND FIGURES**

| **Supplemental Table 1. Clinical characteristics of the FFPE tissue validation cohort** | | | | | | |
| --- | --- | --- | --- | --- | --- | --- |
|  | Benign | PIA | HGPIN | PCI | PCA | PCM |
| **Samples, n** | 10 | 7 | 6 | 7 | 8 | 6 |
| **Mean age, years (range)** | 66.1  (48-79) | 61.3  (49-68) | 61.5  (56-66) | 58  (50-66) | 58.8  (46-69) | 72.9  (59.9-81.4) |
| **Gleason score, n** |  |  |  |  |  |  |
| 6 | - | - | 4 | 7 | - | - |
| 7(3+4) | - | 4 | - | - | - | - |
| 7 (4+3) | - | 3 | 1 | - | 5 | - |
| 8 | - | - | - | - | 3 | 2 |
| 9 | - | - | 1 | - | - | 3 |
| 10 | - | - | - | - | - | 1 |
| **Mean PSA levels, ng/ml (range)** | - | 7.7  (3.2-9.9) | 8.05  (5-11.6) | 5.5  (3.6-7.1) | 8  (4.5-13.6) | 62.5*  (41-105) |
| Data was obtained at diagnosis. * PSA levels missing for 2/6 PCM patients. Clinicopathological features: **Benign** – Obtained at radical cystoprostatectomy and TURP with no evidence of PCa, HGPIN or prostatitis; **PIA** – Obtained at radical prostatectomy with no adjacency to tumour focus; **HGPIN** – Obtained at radical prostatectomy with no adjacency to tumour focus; **PCI** – Obtained at radical prostatectomy: gleason score 6, organ-confined (pT2), pre-operative PSA < 10ng/ml, min. 5 year follow-up with no evidence of biochemical or clinical recurrence; **PCA** – Obtained at radical prostatectomy: primary gleason grade 4 or 5, non-organ-confined disease (pT3), evidence of biochemical recurrence; **PCM** – Obtained at rapid autopsy: various soft tissue metastases arising from lethal castration-resistant PCa. Abbreviations: PIA – Proliferative inflammatory atrophy; HGPIN – High-grade prostatic intraepithelial neoplasia; PCI – Prostate cancer (indolent); PCA – Prostate cancer (aggressive); PCM – Prostate cancer (metastatic). | | | | | | |

**Supplemental Figure 1. Genetic characteristics of the most variable probes across subjects in the study cohort**

Stacked bar graph and table depicting the distribution of the top 5% most variable probes for each patient, based on their location across the genome. Probes located in the 5’ region (range: 9,549 – 11,719) were selected for further analysis. Abbreviations: mCRPC – metastatic castration resistant prostate cancer; mPCa - metastatic prostate cancer.

**Supplemental Figure 2**. **Longitudinal methylation dynamic patterns observed in mPCa patient S002**

**A)** Disease course (since recruitment to iPROSPECT study - baseline), with subject ID indicated in the upper left corner of each graph. PSA levels (ng/ml) and cfDNA concentration (ng/ul) are represented in the left (black) and right (red) y axis, respectively. Details of therapies administered are indicated by coloured lines below the graph: LHRH agonists/antagonist (orange), taxane (pink), AR inhibitors (blue) and radioisotopes (yellow). Red dashed arrow indicates missing value for cfDNA. **B)** Heatmap showing the similarity of patterns between probes, located in 5’ regulatory regions, across time. Probes with similar methylation patterns were aggregated into clusters, which are identified in the heatmap by blue boxes and numbers (dashed line at the top indicates threshold applied to select clusters). Unsupervised clustering was performed using euclidean distances between probes and coloured legend reflects magnitude of those distances (yellow to blue – small to large distance). **C)** Methylation dynamics observed for genes identified through cluster analysis, with total number of genes used indicated in the top right corner for each cluster. Blue filled area represents methylation values observed at each time point, with edges indicating the maximum and minimum values observed. Darker blue line represents the observed mean methylation value of all genes. Black lines, on the upper part of the plots, indicate the duration of administration of a specific PCa therapies, whose effect was explored in our study (taxanes – T; AR inhibitors – AR). Abbreviations: BL – Baseline; cfDNA – cell-free DNA; FU – follow-up; PSA – prostate specific antigen; NTS – new treatment strategy.

**Supplemental Figure 3**. **Longitudinal methylation dynamic patterns observed in mPCa patient S013**

(See Supplemental Figure 2 legend for figure description)

**Supplemental Figure 4**. **Longitudinal methylation dynamic patterns observed in mPCa patient S017**

(See Supplemental Figure 2 legend for figure description)

**Supplemental Figure 5**. **Longitudinal methylation dynamic patterns observed in mPCa patient S020**

(See Supplemental Figure 2 legend for figure description)

**Supplemental Figure 6**. **Longitudinal methylation dynamic patterns observed in mPCa patient S029**

(See Supplemental Figure 2 legend for figure description)

**Supplemental Figure 7**. **Longitudinal methylation dynamic patterns observed in mPCa patient S007**

(See Supplemental Figure 2 legend for figure description)

**Supplemental Figure 8**. **Longitudinal methylation dynamic patterns observed in mPCa patient S008**

(See Supplemental Figure 2 legend for figure description)

**Supplemental Figure 9**. **Longitudinal methylation dynamic patterns observed in mPCa patient S009**

(See Supplemental Figure 2 legend for figure description)

**Supplemental Figure 10. Inverse methylation changes are consistent with a transient methylation shift during taxane administration**

Proportion of probes with a change in methylation after taxane therapy cessation. Total number of probes, per subject, was obtained by assessing changes (gain or loss of methylation) after start of taxane administration. These probes were then analysed to understand if their methylation change would be reversed when therapy administration stopped. These changes were accounted for if there was a methylation change ≥0.1 in the opposite direction of the one observed when therapy was being administered.

**Supplemental Figure 11. Progressive methylation changes observed in differentially methylated PCa genes**

Schematic methylation map for *APC*, *RASSF1* and *EPN1* at several stages of PCA carcinogenesis*.* Each dot represents a single individual and β-values indicated represent a mean value from a number of probes (indicated in the y axis). As not all groups were normality distributed, a Kruskal-Wallis test was used to investigate if there were any differences between the groups, followed by a Dunn’s multiple comparison test to understand were those differences were arising from. *P*-values are as follow: * *P*≤0.05, ** *P*≤0.01, *** *P*≤0.001, **** *P*≤0.0001. Abbreviations: B– Benign; PIA – Proliferative inflammatory atrophy; HGPIN – High-grade prostatic intraepithelial neoplasia; PCI – Prostate cancer (indolent); PCA – Prostate cancer (aggressive); PCM – Prostate cancer (metastatic).

**Supplemental Figure 12**. **Quality control for paired samples in study cohort**

SNP heatmap, using the available 65 control SNPs on the HM450K BeadChip array. Each subject’s serial samples (baseline and follow-ups) cluster together, evidencing no sample mix-ups on this design. Legend indicates methylation level for each SNP (values range from 0-1).
